# Mir-675-5p supports hypoxia-induced drug resistance in colorectal cancer cells

**DOI:** 10.1186/s12885-022-09666-2

**Published:** 2022-05-20

**Authors:** Chiara Zichittella, Maria Magdalena Barreca, Aurora Cordaro, Chiara Corrado, Riccardo Alessandro, Alice Conigliaro

**Affiliations:** 1 Department of Biomedicine, Neuroscience and Advanced Diagnostics (Bi.N.D.), Section of Biology and Genetics, University of Palermo, 90133 Palermo, Italy; 2 Department of Biological, Chemical and Pharmaceutical Sciences and Technologies (STEBICEF), University of Palermo, 90128 Palermo, Italy; 3 Institute for Biomedical Research and Innovation (IRIB), National Research Council (CNR), 90146 Palermo, Italy

**Keywords:** Colorectal cancer (CRC), Hypoxia, MicroRNA, Drug resistance, Apoptosis, 5-fluorouracil (5-FU)

## Abstract

**Background:**

The uncontrolled proliferation of cancer cells determines hypoxic conditions within the neoplastic mass with consequent activation of specific molecular pathways that allow cells to survive despite oxygen deprivation. The same molecular pathways are often the cause of chemoresistance. This study aims to investigate the role of the hypoxia-induced miR-675-5p in 5-Fluorouracil (5-FU) resistance on colorectal cancer (CRC) cells.

**Methods:**

CRC cell lines were treated with 5-Fu and incubated in normoxic or hypoxic conditions; cell viability has been evaluated by MTT assay. MiR-675-5p levels were analysed by RT-PCR and loss and gain expression of the miRNA has been obtained by the transfection of miRNA antagomir or miRNA mimic. Total protein expression of different apoptotic markers was analysed through western blot assay. MirWalk 2.0 database search engine was used to investigate the putative targets of the miR-675-5p involved in the apoptotic process. Finally, the luciferase assay was done to confirm Caspase-3 as a direct target of the miR-675-5p.

**Results:**

Our data demonstrated that hypoxia-induced miR-675-5p counteracts the apoptotic signal induced by 5-FU, thus taking part in the drug resistance response. We showed that the apoptotic markers, cleaved PARP and cleaved caspase-3, increased combining miR-675-5p inhibition with 5-FU treatment. Moreover, we identified pro-caspase-3 among the targets of the miR-675-5p.

**Conclusion:**

Our data demonstrate that the inhibition of hypoxia-induced miR-675-5p combined with 5-FU treatment can enhances drug efficacy in both prolonged hypoxia and normoxia, indicating a possible strategy to partially overcome chemoresistance.

**Supplementary Information:**

The online version contains supplementary material available at 10.1186/s12885-022-09666-2.

## Background

Colorectal cancer (CRC) is the third most common cancer worldwide and the second cause of death worldwide [1, 2]. It is a multifactorial disease caused by a complex pattern of environmental, genetic, and biochemical factors whose risk factors are age, obesity, smoking, and alcohol [3, 4].

Nowadays, CRC treatments allow overall survival of up to 3 years, in the case of advanced disease; these include surgery and radio- and/or chemotherapy. Currently, the most used chemotherapeutic molecule to treat CRC is the 5-fluorouracil (5-FU), a synthetic fluorinated pyrimidine analogue that, along with many other cytotoxic drugs, has a strong toxicity-exposure relationship. Moreover, despite the advances in therapeutic plans, the five-year survival of CRC patients remains too low due to the occurrence of innate or acquired drug resistance [5–7]. To treat advanced CRCs, it is required to integrate 5-fluorouracil (5-FU) based therapy with oxaliplatin, reaching a drug-response rate of around 50% [8, 9]. The acquisition of chemotherapy resistance is a complex process whose underlying mechanism has not been fully elucidated. Cancer drug resistance in fact, can occur through different strategies, including cell death inhibition (apoptosis suppression), alteration in drugs’ metabolism, epigenetic and drug targets, enhanced DNA repair and gene amplification [10].

Among the identified mechanisms responsible for 5-FU resistance are the alterations in enzymes involved in the drug’s metabolism, such as the increase of thymidylate synthase activity [11], and the dysregulation of drug transporters that induces multidrug resistance (MDR) [12, 13]. In addition, cellular processes as apoptosis, autophagy and cell cycle could be altered in CRC cells thus affecting response to 5-FU therapy [7, 14–16].

Another mechanism that can induce chemo-resistance is the low oxygen tension (hypoxia) established in growing tumour masses. The hypoxic microenvironment can promote tumour progression by inducing cell cycle deregulation and by allowing apoptosis escape; it has been associated with a poor prognosis for many cancers including breast [17], hepatocellular carcinoma (HCC) [18] and CRC [19].

The hallmark of the cellular responses to low oxygen partial pressure is the stabilization of the hypoxia-inducible factor 1α (HIF-1α) that, migrating in the nucleus and activating its target genes, induces molecular and phenotypic changes promoting cell survival, plasticity, motility and resistance to several anticancer drugs, including 5-FU. To date, numerous attempts to inhibit HIF activity for the treatment of solid tumours failed to meet the expectations, presumably due to the pleiotropism of this transcription factor [20]. Hence the need to understand the molecular mediators through which HIF determines the more aggressive phenotype and chemoresistance, to identify new and effective therapeutic targets.

Over the past few decades, many studies indicated the relevance of non-coding RNAs (ncRNAs) in hypoxia-driven cancer progression and correlated their overexpression with poor prognosis [21]. Recently, it was shown that hypoxia-induced ncRNAs LUCAT1 confers chemoresistance to CRC cells both in vitro and in vivo. LUCAT1 physically interacts with PTBP1 (Polypyrimidine Tract Binding Protein 1) to modulate the alternative splicing of a set of DNA damage-related genes [22].

Among the hypoxia-induced ncRNA is the long non-coding H19 (lncH19) [23, 24] whose increase convey with the selective increase of its intragenic miR-675-5p [25–28]. As hypoxia-induced ncRNA the miR-675-5p maintain hypoxic responses by controlling HIF1α mRNA stability [26] and, in CRC, it modulates tumor progression by regulating HIF1α-induced EMT (epithelial-mesenchymal transition) [25]. Moreover, our recent paper showed that hypoxia-induced miR-675-5p supports β-catenin nuclear localization by regulating GSK3-β activity in CRC cell lines [28]. To the best of our knowledge, few data demonstrated a direct correlation between hypoxia-induced ncRNAs and chemoresistance and in particular the role of the miR-675-5p in hypoxia-induced drug resistance has not been yet investigated.

This study aims to investigate the role of the hypoxia-induced ncRNA miR-675-5p in 5-FU chemoresistance on CRC cells, to identify new and more effective molecular targets for the treatment of colorectal cancer.

## Methods

### Cell culture

HCT116 and SW480 cells (ATCC-LGC Standards S.r.L., Italy) were cultured respectively in McCOY’S 5A medium and RPMI 1640 (Euroclone, UK) supplemented with 10% Fetal Bovine Serum, 1% Penicillin/Streptomycin (10,000 U/mL Penicillin and 10 mg/mL Streptomycin) and 200 mM L-Glutamine (all from Euroclone, UK).

Cells were maintained in a humidified atmosphere of 5% CO_2_ at 37 °C and used at early passages (under 10 passages) for all experiments. The culture medium was changed every 2-3 days, and cells were split at 70–80% of confluence.

### Hypoxia assay

To perform hypoxia experiments, cells were seeded on cell culture plates (Sarstedt), maintained for 24 hours in a humidified atmosphere of 5% CO_2_ at 37 °C, and finally moved into a hypoxic chamber (Stemcell™ Technologies, Voden Medical Instruments spa, Italy) containing 1% O_2_ gas mixture for 72 hours, the suitable time to achieve hypoxia-induced drug resistance [18].

### MTT (3-[4,5-Dimethylthiazol-2-yl]-2,5 diphenyl tetrazolium bromide) assay

Cell viability was determined by MTT assay following the manufacturer’s instructions (Cat. n° M6494, Thermo Fisher®, USA) and the absorbance at 540 nm was measured by the Microplate Reader (BioTek Instruments, USA) .

HCT116 and SW480 were seeded in quadruplicate respectively at 3 × 10^4^ or 2.5 × 10^4^ cells/cm^2^. After 24 hours, the cells were treated with 5 or 10 μM of 5-FU (5-Fluorouracil, cat. n° F 6627, Sigma-Aldrich, St. Louis, MO, USA) and placed for 72 hours in hypoxic (hypoxic chamber containing 1% O_2_ gas mixture) or normoxic conditions.

### Transfection

HCT116 and SW480 cells were seeded respectively at 3 × 10^4^ or 2.5 × 10^4^ per cm^2^. The day after, cells were transfected with 3.7 pMoles/cm^2^ of miRCURY LNA miRNA Inhibitor hsa-miR-675-5p (Cat. n°339,203 YCI0202815-FZA, Qiagen, Germany), or miRCURY LNA miRNA Inhibitor Negative Control (Cat. n°339,203 YCI0202036-FZA, Qiagen, Germany). For cell transfection, HiPerFect Transfection Reagent (Cat. n° 301,704, Qiagen, Germany) was used following the manufacturer’s standard instructions. Six hours after transfection, the cells were treated with 10 μM of 5-FU in a fresh medium and placed for 72 hours in hypoxic or normoxic conditions.

After this time cells were used for MTT assay or protein and RNA extraction.

### RNA extraction and real-time PCR (RT-PCR)

Total RNA was extracted using the commercially available TRIzol® RNA Isolation Reagents (Cat. n° 15,596,026, Thermo Fisher® Products & Kits, USA) according to the manufacturer’s instructions. The total RNA concentration was detected with Nanodrop spectrophotometer (Thermo Fisher®, USA). Reverse transcription and qRT-PCR were performed following the manufacturer’s instruction by the use of TaqMan™ MicroRNA Reverse Transcription Kit (Cat. n° 4,366,596, Applied Biosystem™, USA) and TaqMan™ Fast Universal PCR Master Mix. (Cat. n° 4,352,042, Applied Biosystems™, USA). For probes and oligonucleotides were used Has-miR-675-5p cod. TM002005 and U6 snRNA cod. TM001973 (all from Applied Biosystems™, USA). Hsa-miR-675-5p expression levels were normalized to U6 snRNA and data are presented as 2^-ΔΔCt.

### MirWalk target prediction

The miR-675-5p targets prediction among apoptosis pathay was performed using the tool Target Mining of mirWalk 2.0 database search engine [29].

### Western blotting

HCT116 and SW480 cells were lysed for 1,30 hours in Lysis Buffer (15 mM Tris/HCl pH 7.5, 120 mM NaCl, 25 mM KCl, 1 mM EDTA, 0.5% Triton X100) addicted with Phosphatase Inhibitor cocktail (Cat. n° 37,492, Active Motif, USA). Cell debris was removed by centrifugation at 17.000 g for 15′ at 4 °C and the supernatant, containing protein lysate, was quantified by the Bradford microassay method (Pierce™ Coomassie Plus Assay Kit, Cat. n° 23,236, ThermoFisher Scientific, USA) using Bovine Serum Albumin (BSA, Cat. n° A2153, Sigma-Aldrich, USA) as a standard. A total of 15 μg of protein from each sample was separated using Bolt Bis-Tris gel 4 – 12% (Cat. n° NP0326BOX, ThermoFisher Scientific, USA) and transferred on nitrocellulose membranes with pore size 0.45 μm (Cat. n° GEH10600002, GE Healthcare, USA). The membranes were coloured with 0.1% Rosso Ponceau in 5% acetic acid to evaluate the correct loading and migration of all samples. The membranes were incubated for 1 hour in blocking solution (5% BSA, 20 mM Tris, 140 mM NaCl, 0.1% Tween-20) and overnight with the primary antibodies: anti-Carbonic Anhydrase/CA9 (1:1000, Cat. n° 5648S, Cell Signaling Technology, USA), anti-PARP-1 (1:500, Cat. n° sc-8007, Santa Cruz Biotechnology USA), anti-Cleaved Caspase-3 (1:400, Cat. n° 9664S, Cell Signaling Technology, USA), anti-Caspase-3 (1:500, Cat. n° sc-7272, Santa Cruz Biotechnology, USA), anti-Caspase-9 (1:750, Cat. n° 9502, Cell Signaling Technology, USA), and anti-β-Actin (1:1500, Cat. n° sc-81,178, Santa Cruz Biotechnology, USA). After five washes in TBST buffer (20 mM Tris, 140 mM NaCl, 0.1% Tween-20) the membranes were incubated with appropriate secondary antibody HRP, Goat anti-Rabbit IgG (1:10.000, Cat. n° 31,460, Invitrogen, Thermo Fisher® Scientific, USA) and anti-mouse IgG (1:10.000, Cat. n° 7076, Cell Signaling Technology, USA). The chemiluminescent signal was detected by the Chemidoc acquisition instrument (Bio-Rad, USA). The obtained images were analyzed with the Image Lab software (Bio-Rad, USA). If required, depending on protein molecular weight, the membranes were subjected to stripping protocol, before proceeding with further staining. Briefly, 15′ incubation with stripping solution (Restore™ PLUS Western Blot Stripping Buffer, Cat. n° 46,430, Thermo Fisher® Scientific, USA) at 37 °C.

### Firefly luciferase assay

For validation of Pro-Caspase 3 as a target of miR-675-5p, HCT116 cells were seeded at 7 × 10^4^ cells/cm^2^ and 24 hours after seeding, transfected with Attractene Transfection Reagent (Cat. n° 301,005, Qiagen, Germany) for 24 hours with 100 ng (3.7 pMoles/cm^2^) of mirVana™ hsa-miR-675-5p mimic (Mimic miR-675-5p, Assay ID MC12067, Thermo Fisher®, USA) or mirVana™ Scrambled Negative Control (Scr) and with 50 ng of Reporter plasmid DNA (Caspase-3 Human 3′ UTR Clone/RFP, Cat. n° SC215501, OriGene Technologies, Inc) used following the manufacturer’s standard application guide. Then 24 hours after transfection, luciferase tests were performed using the Firefly Luciferase Assay Kit (Cat. # PR300001, OriGene Technologies, Inc) following the manufacturer’s standard instructions. Luminescence and fluorescence were detected by GloMax®-Multi Microplate Reader (Promega, USA). The luminescence was normalized for the Red Fluorescent Protein (RFP) values and the relative Luciferase activity following the overexpression of the hsa-miR-675-5p mimic (Luc/RFP + Mimic miR-675-5p) is expressed in fold change with respect to the Negative Control (Luc/RFP + Scr).

### Statistical analysis

Data are reported as mean ± standard deviation (SD) of at least three biological replicates. Statistical analyses: Student’s t-test or Ordinary one-way ANOVA with Bonferroni’s multiple comparison test were performed by using GraphPad Prism software (GraphPad Software, USA). *P*-values were indicated in the graphs as follow: * = *p <* 0.05; ** = *p <* 0.01; *** = *p <* 0.001; **** = *p <* 0.0001.

## Results

### Prolonged hypoxia induced chemo-resistance to the 5-FU treatment and enhanced miR-675-5p expression

**Fig. 1:**
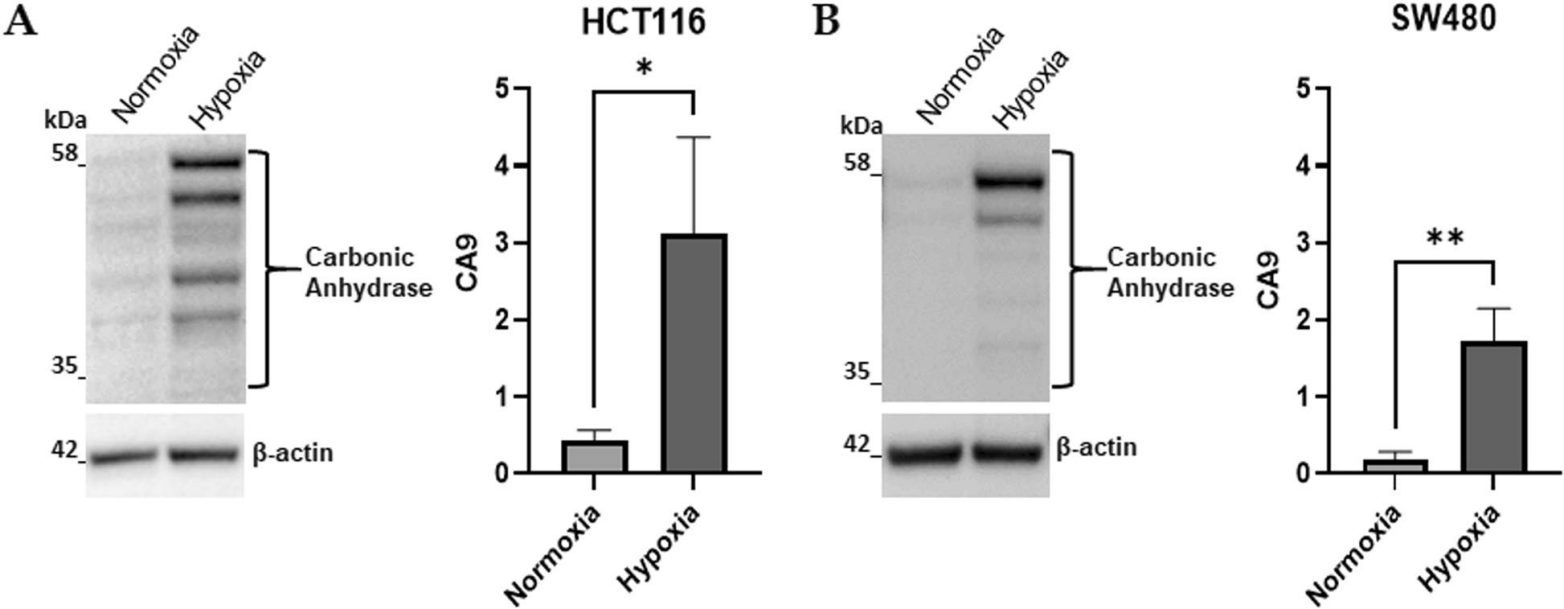
Carbonic Anhydrase 9 expression confirms the hypoxic condition. **A-B**: Representative images and densitometric analysis of Western blots for Carbonic Anhydrase (CA9) obtained from protein lysates of HCT116 and SW480 in normoxic conditions or subjected to hypoxic conditions. The graphs ordinate shows the OD (Optical Density) of the indicated proteins normalized for the housekeeping’s OD (β-actin). Data are expressed as the mean ± SD of three independent experiments and statistical significance was analyzed using a Student’s t-test (* = *p <* 0.05; ** = *p <* 0.01). Corresponding uncropped full-length blots are included in Supplementary Materials

Long-time exposure to hypoxic conditions, beyond 48 hours, is known to activate molecular pathways leading cancer cells to promote survival strategies including chemo-resistance [30–32]. To reproduce this condition in vitro, CRC cell lines (HCT116 and SW480) were treated with different concentrations of 5-FU and maintained in a hypoxic chamber containing 1% O_2_ gas mixture for 72 hours. The activation of hypoxic response in our model was confirmed by the increase of the carbonic anhydrase 9 (CA9), a primary HIF’s target [33] (Fig. 1) [32, 33].

**Fig. 2:**
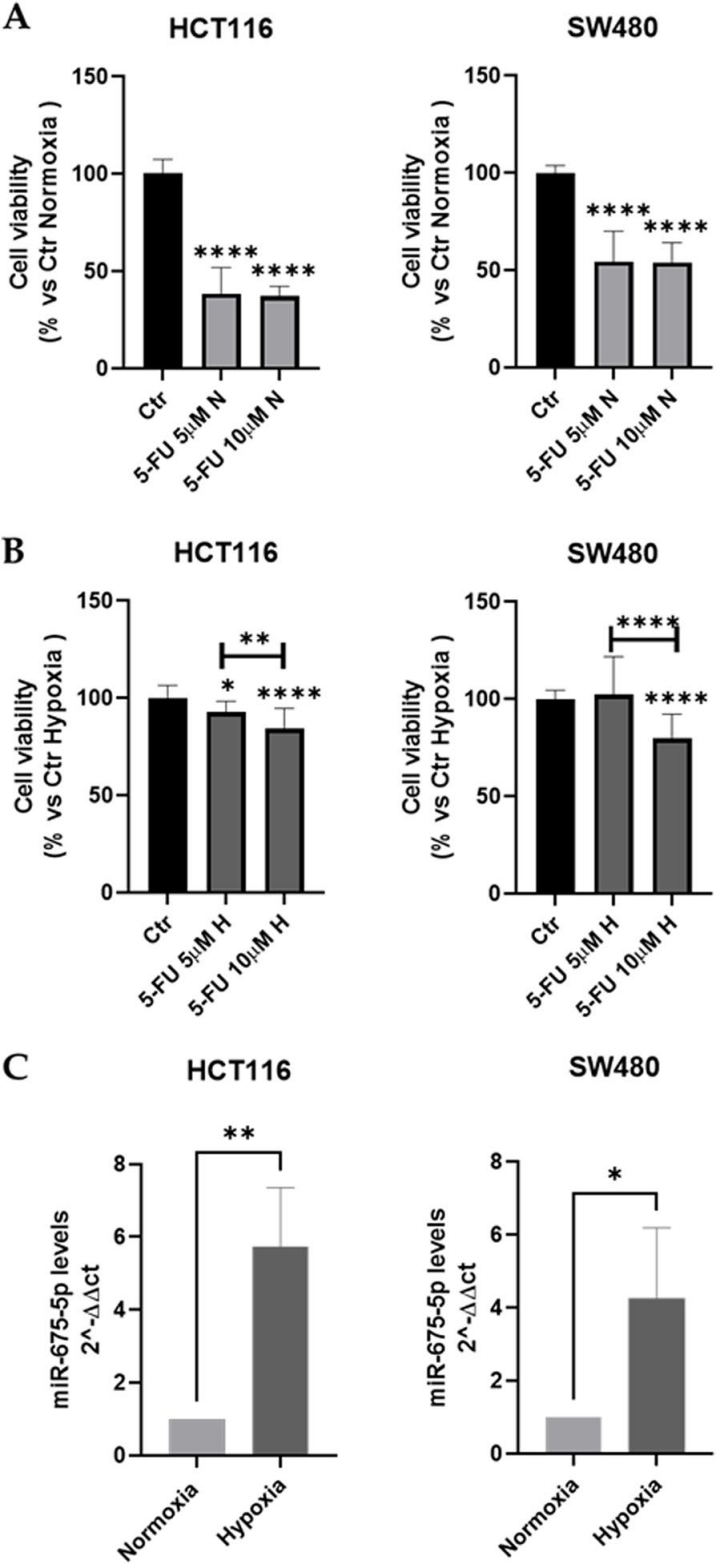
Colon cancer cell lines behaviour under chronic hypoxic stimulation (72 h). **A-B**: Cell viability assay (MTT Assay) in HCT116 and SW480 treated for 72 h with two different concentrations of 5-FU (5 μM and 10 μM) in normoxic (N) conditions or subjected to hypoxic (H) conditions. Data are expressed as the percentage of cell viability versus untreated cells both in normoxia and chronic hypoxia (Ctr). **C**: Analysis of the expression level (qRT-PCR) of miR-675-5p in HCT116 and SW480 under normoxic conditions and after 72 hours of hypoxic stimulation. The miR-675-5p levels were normalized for RNU6 (U6 Small Nuclear 1), and the ΔΔCt was calculated with respect to the expression levels under normoxic conditions. All data are the mean ± SD of three biological replicates. Statistical analyses: Ordinary one-way ANOVA with Bonferroni’s multiple comparison test were used in Figs. A and B, Student’s t-test was used for Fig. C (* = *p <* 0.05; ** = *p <* 0.01; **** = *p <* 0.0001)

To investigate the effects of hypoxia on cell survival, MTT assays have been done. As expected, the cell viability assay showed that 5-FU treatments induced cell death in CRC cell lines in normoxic conditions while it did not occur in hypoxic conditions (Fig. 2A-B). These data supported the use of our model as a tool to investigate the molecular mechanisms controlling hypoxia-induced chemoresistance. Further experiments have been performed by using the higher dose of 5-FU.

Our previous manuscripts identified the miR-675-5p as hypoxia-induced miRNA with a role in mediating acute hypoxic responses. However, the expression of miR-675-5p after prolonged hypoxic stimulation has not been yet investigated [25, 28]. The RT-PCR in Fig. 2C revealed that CRC lines after prolonged hypoxia (72 hours) express higher levels of miR-675-5p compared to cells in normoxic conditions. These data prompted us to investigate its role in drug resistance.

### The use of miR-675-5p antagonist counteracted the hypoxia-induced drug resistance

**Fig. 3:**
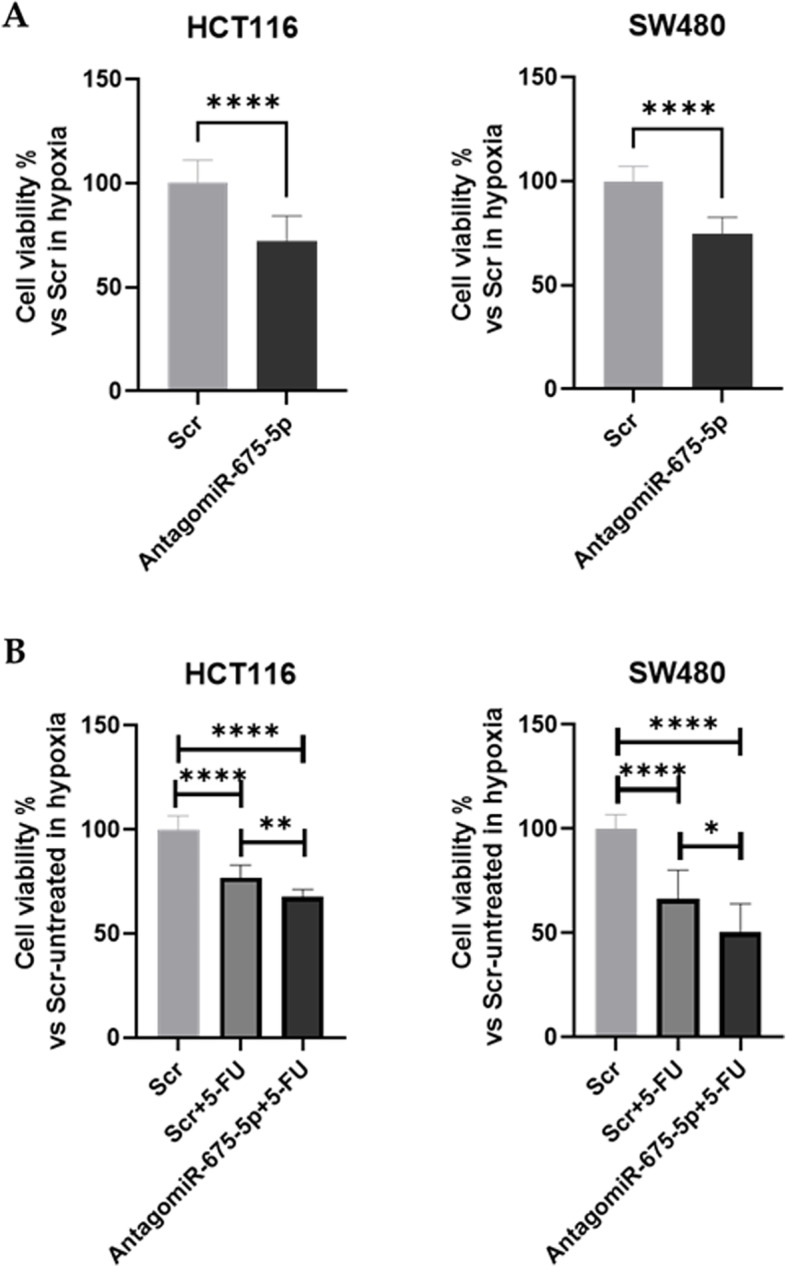
Effects of AntagomiR-675-5p treatment in cell viability in chronic hypoxic conditions. **A**: Cell viability assay (MTT Assay) in HCT116 and SW480 transfected with AntagomiR-675-5p or Scrambled Negative Control (Scr) and grown in the hypoxic chamber for 72 h. Data are expressed as cell viability percentage compared to cells transfected with Scr. **B**: Cell viability assay (MTT Assay) in HCT116 and SW480 transfected with AntagomiR-675-5p or Scramble Negative Control (Scr) and treated or not for 72 h of hypoxia with 5-FU (10 μM). Data are expressed as the mean ± SD of three biological replicates. Statistical analyses: Student’s t-test was used for Fig. A, Ordinary one-way ANOVA with Bonferroni’s multiple comparison test were used in Fig. B (* = *p <* 0.05; ** = *p <* 0.01; **** = *p <* 0.0001)

Firstly through miRNA inhibition, we explored the role of hypoxia-induced miR-675-5p in cell viability. MTT assay revealed that in both cell lines, treatment with miRNA AntagomiR-675-5p reduced cell viability of hypoxic cells (Fig. 3A). In the light of this, we investigated whether treatment with AntagomiR-675-5p could enhance the effect of 5-FU thus overcoming the hypoxia-induced chemoresistance.

The cell viability assay confirmed our hypothesis indicating that, in hypoxic conditions, cells treated with both 5-FU and AntagomiR-675-5p showed a higher reduction of cell viability, compared to cells treated with the drug alone (Fig. 3B).

**Fig. 4:**
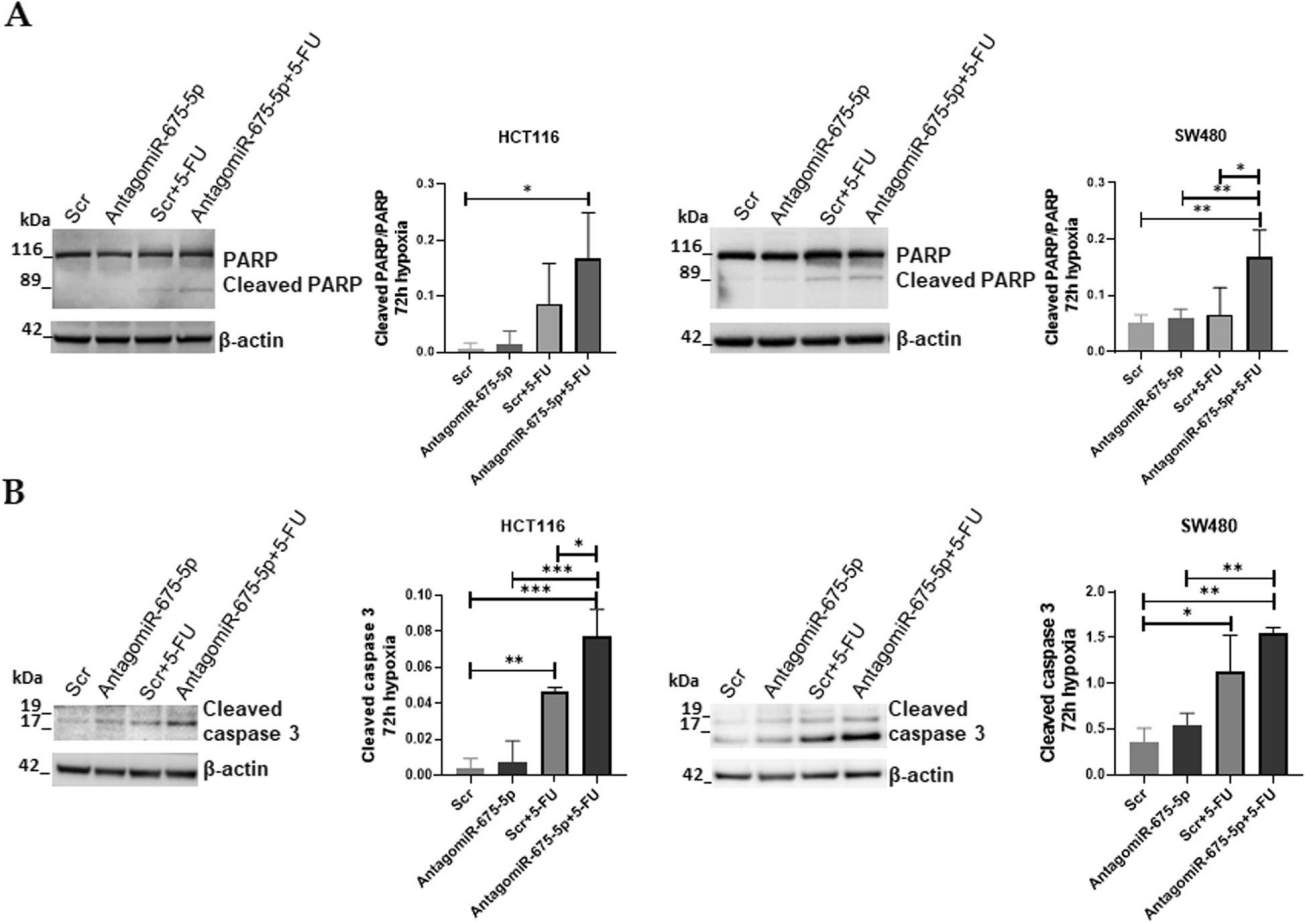
Effects of AntagomiR-675-5p treatment on apoptosis markers. **A-B**: Representative images and densitometric analysis of Western blots for cleaved PARP/PARP and Cleaved caspase-3 obtained from protein lysates of HCT116 and SW480 in chronic hypoxia, transfected with AntagomiR-675-5p or Scrambled Negative Control (Scr) and treated or not with 5-FU (10 μM). The graphs ordinate shows the OD (Optical Density) of the indicated proteins normalized for the housekeeping’s OD (β-actin). Data are expressed as the mean ± SD of three independent experiments and statistical significance was analyzed by using Ordinary one-way ANOVA with Bonferroni’s multiple comparison test (* = *p <* 0.05; ** = *p <* 0.01; *** = *p <* 0.001; **** = *p <* 0.0001). Corresponding uncropped full-length blots are included in a Supplementary Material

It is known that 5-FU treatment in CRC promotes apoptosis through caspase-9 activation [34], here we investigated if the addition of the AntagomiR-675-5p promotes cell death by enforcing cell entrance into apoptosis. To this aim, western blot analyses for apoptotic markers were done in hypoxic cells (1% O_2_ gas mixture) transfected with AntagomiR-675-5p or Scrambled Negative Control (Scr) and treated or not with 5-FU (10 μM). As shown in Figs. 4A-B the treatment with 5-FU induced PARP cleavage and increased the levels of cleaved caspase-3, interestingly these effects were further improved by the addition of AntagomiR-675-5p to the drug. Overall these data indicated a role for the miR-675-5p in inhibiting apoptosis.

### MiR-675-5p directly targeted caspase 3 3’UTR

**Fig. 5:**
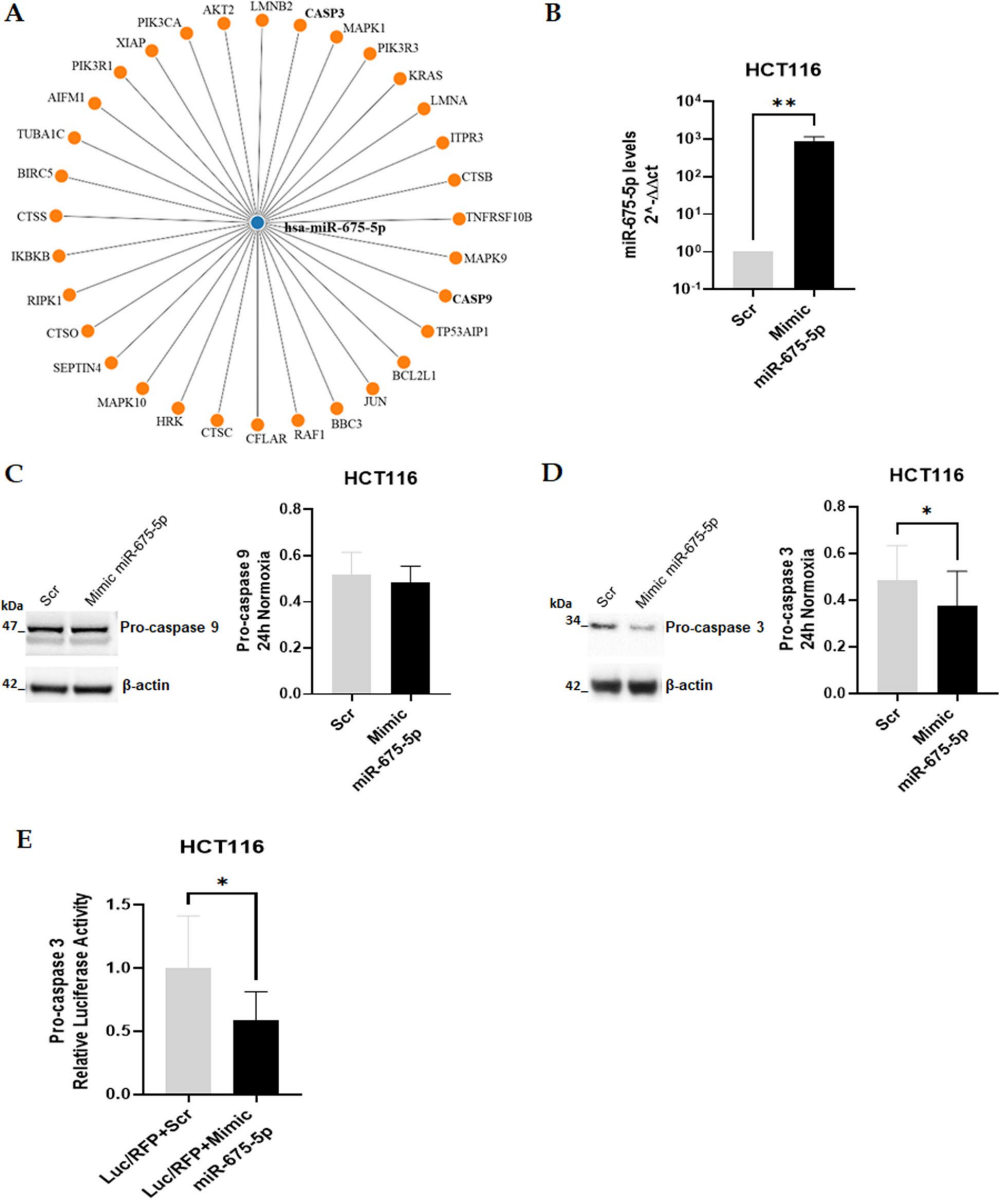
Identification of miR-675-5p targets involved in apoptosis. **A**: The network diagram obtained using the mirWalk database [29] illustrates the presumed 3’UTR targets of miR-675-5p involved in apoptosis (KEGG Pathway hsa04210#Apoptosis). **B**: Expression level analysis (qRT-PCR) of miR-675-5p in HCT116 after overexpression of miR-675-5p under normoxic conditions. The miR-675-5p levels were normalized for RNU6 (U6 Small Nuclear 1), and the ΔΔCt was calculated with respect to the Scrambled Negative Control (Scr). **C-D**: Representative images and densitometric analysis of Western blots respectively for pro-caspase 9 and pro-caspase 3 on proteins lysates from HCT116 transfected with miR-675-5p mimic or Scrambled Negative Control (Scr) for 24 h in normoxia. The graphs ordinate shows the OD (Optical Density) of the indicated proteins normalized for the housekeeping’s OD (β-actin). Corresponding uncropped full-length blots are included in a Supplementary Materials. **E**: The Firefly Luciferase assay validates pro-caspase 3 as the target of miR-675-5p. Luminescence was normalized for RFP values end presented in the graph as relative Luciferase activity in cells treated with mimic-miR-675-5p (Luc/RFP + Mimic miR-675-5p) with respect to cells treated with the Negative Control (Luc/RFP + Scr). All Data are expressed as mean ± SD of three independent experiments and statistical significance was analyzed using Student’s t-test (* = *p <* 0.05; ** = *p <* 0.01)

By querying the miRWalk database [29], we obtained the list of the putative miR-675-5p targets involved in apoptosis (Fig. 5A) (KEGG Pathway hsa04210#Apoptosis). Considering the effects shown by the AntagomiR-675-5p in hypoxic conditions we decided to investigate firstly the caspases of the intrinsic apoptosis pathway: caspase-9 and caspase-3. Targets’ validation has been performed only in HCT116. We transfected HCT116 cells with miRNA-675-5p mimic (Fig. 5B) and investigated protein levels of both putative targets. Transfection was performed on cells in normoxia as they express lower levels of miR-675-5p.

The western blot in Fig. 5D indicated that miRNA overexpression in normoxic cells induced a reduction in pro-caspase-3 while no effects have been revealed in pro-caspase-9 (Fig. 5C). The direct targeting of caspase-3 3’UTR, has been further confirmed by Luciferase assay (Fig. 5E).

Overall the data demonstrated that, in HCT116 CRC cells grown in normoxic conditions, AntagomiR-675-5p enforces the pro-apoptotic effects of 5-FU treatment by protecting caspase-3 from miRNA-675-5p mediated inhibition.

**Fig. 6:**
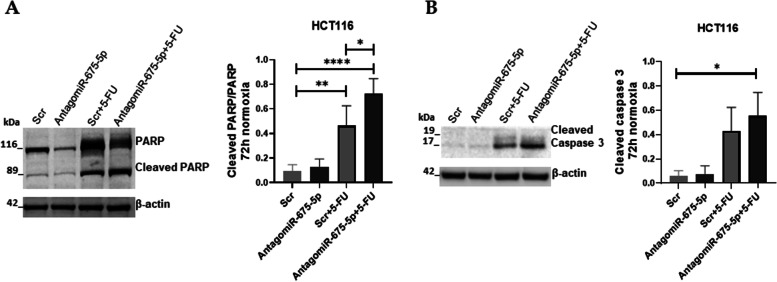
Effects of AntagomiR-675-5p on HCT116 cell line in normoxic conditions. **A-B**: Representative images and densitometric analyses of Western blots for cleaved PARP/PARP and Cleaved caspase-3 in HCT116 in normoxic conditions, transfected with AntagomiR-675-5p or Scrambled Negative Control (Scr) and treated or not with 5-FU (10 μM). The graphs ordinate shows the OD (Optical Density) of the indicated proteins normalized for the housekeeping’s OD (β-actin). Data are expressed as the mean ± SD of three independent experiments and statistical significance was analysed by using Ordinary one-way ANOVA with Bonferroni’s multiple comparison test (* = *p* < 0.05; ** = *p <* 0.01; **** = *p <* 0.0001). Corresponding uncropped full-length blots are included in a Supplementary Material

Finally, we wondered if AntagomiR-675-5p could reinforce the effect of 5-FU even when miR-675-5p concentrations are not as high as in some cases of primary tumor or in cells under normoxic conditions [25]. Figure 6 indicated that, although with less intensity than in hypoxic conditions, in HCT116 cells the use of AntagomiR-675-5p enhanced the apoptotic process induced by the 5-FU whereas AntagomiR-67-5p alone did not affect cell viability, unlike what occurred in hypoxia.

## Discussion

CRC still maintain a leading position among the causes of cancer deaths [1, 2]. Although extensive advances in CRC treatments have been reached, chemoresistance to drug treatment remains the major cause of recurrence and metastasis.

Nowadays it is important to dissect the molecular mechanisms underlying chemoresistance processes, to identify new therapeutic targets and to enhance the action of conventional therapy [5, 6, 10].

Increasing data obtained from experimental and clinical studies have shown that intratumoral hypoxia is a common feature of human cancers contributing to the development of resistance to radiation and chemotherapy [35, 36]. Meanwhile, several studies confirmed the role of hypoxia-induced non-coding RNAs as pivotal players mediating hypoxic responses, including drug resistance [21, 22, 37–40].

Among them, we and others attributed the LncRNA H19 and its intragenic miRNA miR675-5p an important role in promoting cancer onset and progression [23, 26, 41–46]. In CRC it has been demonstrated that lncH19 mediates 5-FU resistance enforcing SIRT1 mediated autophagy [47], while its expression by cancer-associated fibroblasts, promotes stemness and chemoresistance of CRC [48]. Moreover, is through the expression of its intragenic miR-675-5p that lncH19 promotes drug resistance to 1,25-dihydroxyvitamin D3 treatment; since miR-675-5p inhibits the expression of Vitamin D Receptor [43].

Here we demonstrated, for the first time to our knowledge that the miR-675-5p, which expression is markedly increased by the hypoxic microenvironment, enforces drug resistance by affecting 5-FU induced apoptosis through the inhibition of caspase-3.

Resistance to chemotherapy treatment is often caused by processes that inhibit the apoptosis induced by the drug, to overcome this limit several miRNAs have been identified as possible drug co-operators. MiR-206, miR-148a, miR-125a-5p and miR-129 can target BCL2, reducing its anti-apoptotic role and the overexpression of these miRNAs increased the sensitivity of CRC cells to 5-FU [49–52]. MiR-143 increased the sensitivity of colorectal cancer cells to 5-FU stimulated apoptosis by down-regulating BCL-2 and activating caspases 3, 8, and 9 and [53]. Also, miR-182 by inducing caspase-3/PARP, and miR-34a by targeting SIRT1, significantly increase apoptosis in CRC. On the other hand, the reduction of miRNA such as miR-135b, miR-21 and miR-587, involved in apoptosis, can be considered a solution to enhance the apoptosis of CRC cells [54–56].

To verify the possible correlation between miR-675-5p and apoptosis pathways we used the miRWalk database, to obtain a network of the 3’UTR putative targets of this miRNA. We found that miR-675-5p may target many mRNAs involved in apoptosis, such as caspase-3 and caspase-9. Here we have confirmed the binding of miR-675-5p to caspase 3 however other putative markers remain to be investigated.

Moreover, our data indicated that, in prolonged hypoxia, the miR-675-5p may promote cell viability in multiple ways. MTT assay revealed that miR-675-5p inhibition reduced cell viability in hypoxic cells however, treatment with the AntagomiR-675-5p alone showed no cleavage in either Caspase 3 or PARP. Our previous manuscript demonstrated that miR-675-5p inhibition impedes beta-catenin nuclear localization in hypoxic CRC cells inducing inhibition in Cyclin D expression. It is reasonable to assume that this inhibitory effect may be reflected in a slowing of the cell cycle. However, further data must be produced to support this hypothesis.

## Conclusion

**Fig. 7:**
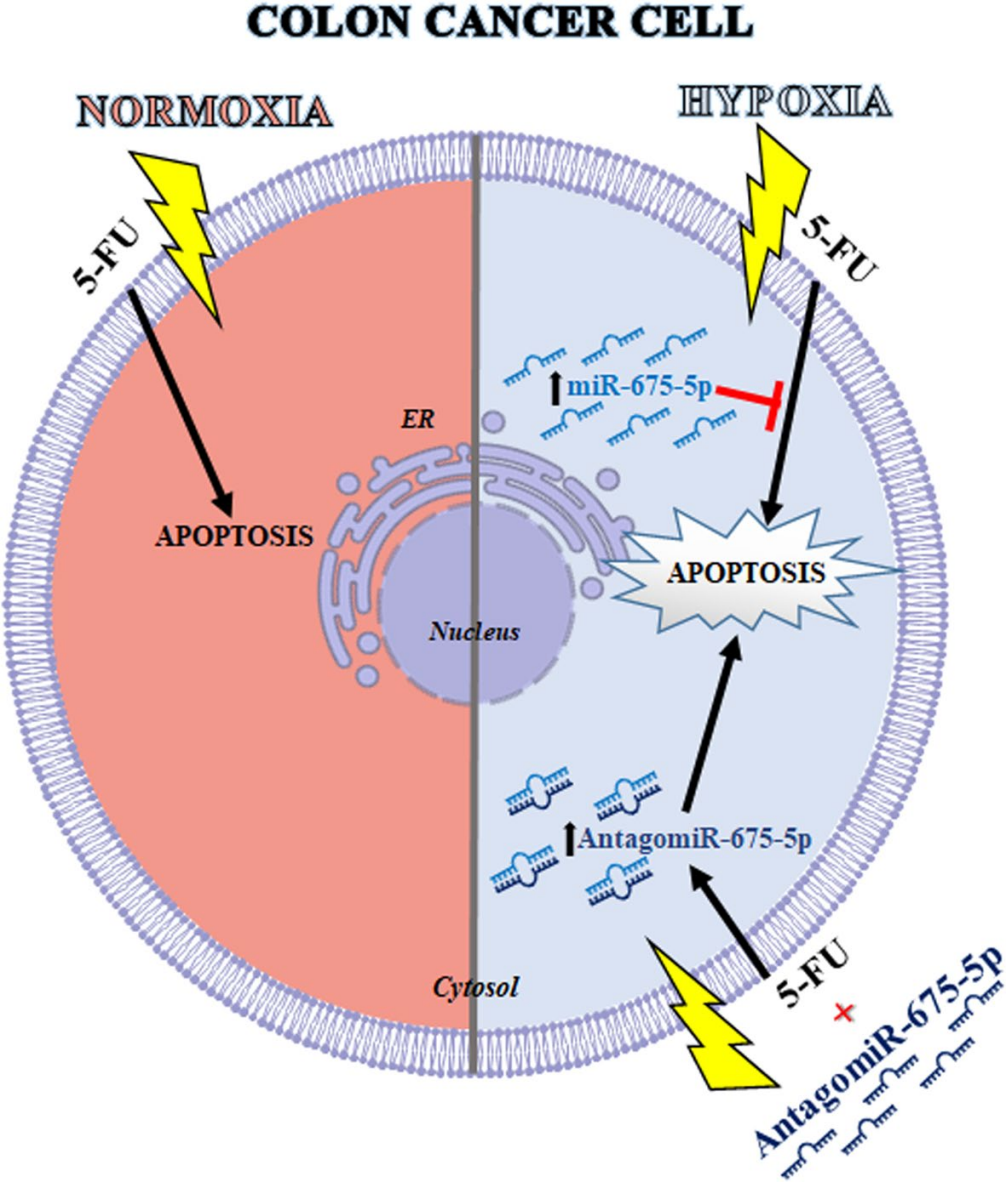
Schematic representation of the proposed model. On the left in red is represented the CRC cell treated with the chemotherapeutic drug 5-Fluorouracil (5-FU) which in normoxic conditions activates the apoptotic process. In blue at the top right is represented the CRC cell treated with 5-FU in conditions of prolonged hypoxia, in which the overexpression of miR-675-5p inhibits the activation of the apoptotic process by targeting the pro-caspase 3. Finally, below on the right in blue is represented the CRC cell treated with 5-FU in conditions of prolonged hypoxia, in which the presence of AntagomiR-675-5p activates the apoptotic process, increasing the protein levels of the cleaved caspase-3 and the cleaved PARP

Our in vitro data unveiled a possible role for the hypoxia-induced miR-675-5p as a promoter of 5-FU drug resistance. We demonstrated that the use of miRNA-675-5p inhibitor in combination with the drug 5-FU could enforce the action of the last, overcoming at least in part a chemo-resistant situation (Fig. 7). Our data suggested that the combined action of the drug and AntagomiR-675-5p could lead to a decrease in therapeutic drug doses, but further in vivo studies are needed to confirm this hypothesis.

## Supplementary Information

**Additional file 1.**

## Data Availability

The datasets used and/or analysed during the current study are available from the corresponding author on reasonable request.

## Abbreviations

CRC: Colorectal cancer
5-FU: 5-Fluorouracil
MDR: Multidrug resistance
HIF-1α: Hypoxia-inducible factor 1α
ncRNA: Non coding RNA
miRNA: microRNA
lncH19: Long non-coding H19
MTT: 3-(4,5-Dimethylthiazol-2-Yl)-2,5-Diphenyltetrazolium Bromide
RT-PCR: Real Time Polymerase Chain Reaction
PBS: Phosphate Buffered Saline
SD: Standard deviation
BSA: Bovine Serum Albumin
CA9: Carbonic Anhydrase 9
RFP: Red Fluorescent Protein
