# Supplementary material for: Mir-675-5p supports hypoxia-induced drug resistance in colorectal cancer cells
Source: BMC Cancer. 2022 May 20;22:567. doi: 10.1186/s12885-022-09666-2 (PMC9123752; doi:10.1186/s12885-022-09666-2)
Supplement: Supplementary file 1 — Additional file 1. [file 12885_2022_9666_MOESM1_ESM.pdf]

Mir-675-5p supports hypoxia-induced drug resistance in colorectal cancer cells

Chiara Zichittella[1], Maria Magdalena Barreca[1,2], Aurora Cordaro[1], Chiara Corrado[1], Riccardo Alessandro[1,3], Alice Conigliaro[1*]

[1] Department of Biomedicine, Neuroscience and Advanced Diagnostics (Bi.N.D.), Section of Biology and Genetics, University of Palermo, 90133 Palermo, Italy

[2] Department of Biological, Chemical and Pharmaceutical Sciences and Technologies (STEBICEF), University of Palermo, 90128 Palermo, Italy

[3] Institute for Biomedical Research and Innovation (IRIB), National Research Council (CNR), 90146 Palermo, Italy

*correspondence: alice.conigliaro@unipa.it

Supplementary Materials

Full-length blots of figure 1

A **HCT116**

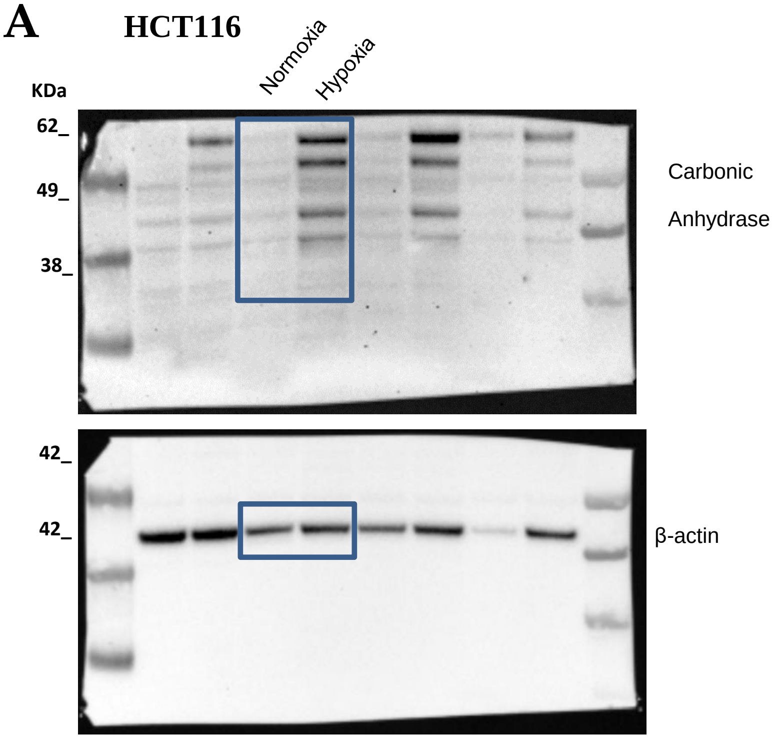

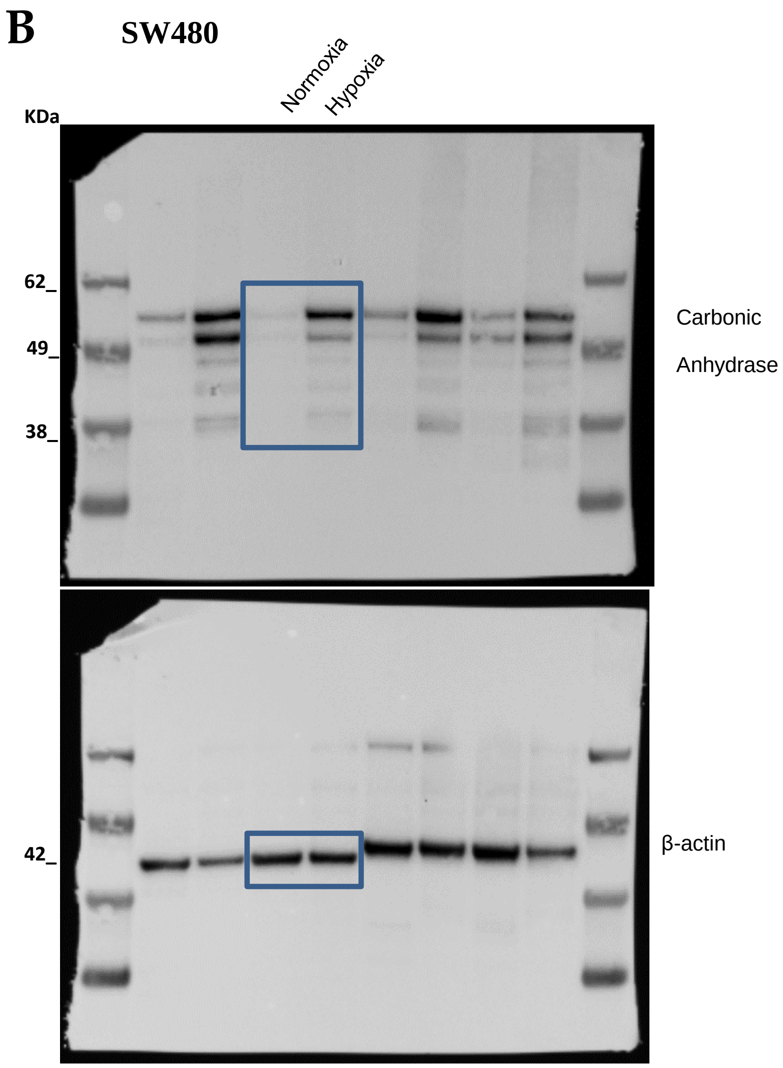

Uncropped full-length blots of figure 1: western blot for Carbonic Anhydrase (CA9) and β-actin of protein lysates of HCT116 (**A**) and SW480 (**B**) under normoxic or hypoxic conditions.

Full-length blot of figure 4

A

HCT116 **SW480**

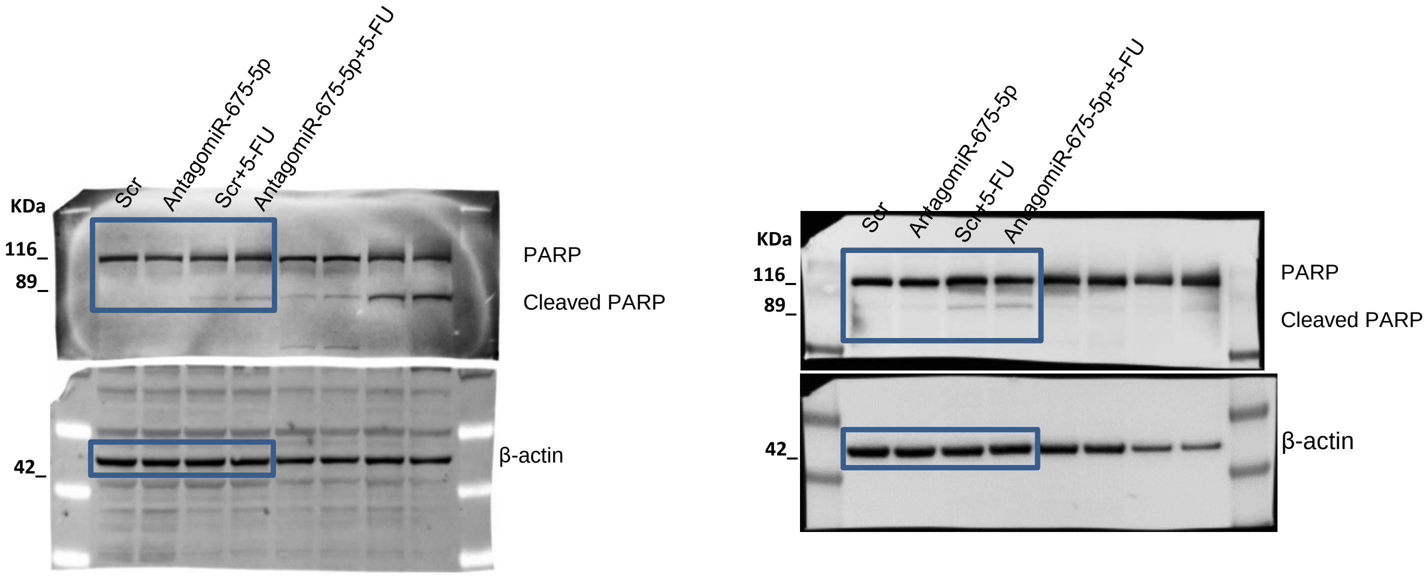

B

HCT116 **SW480**

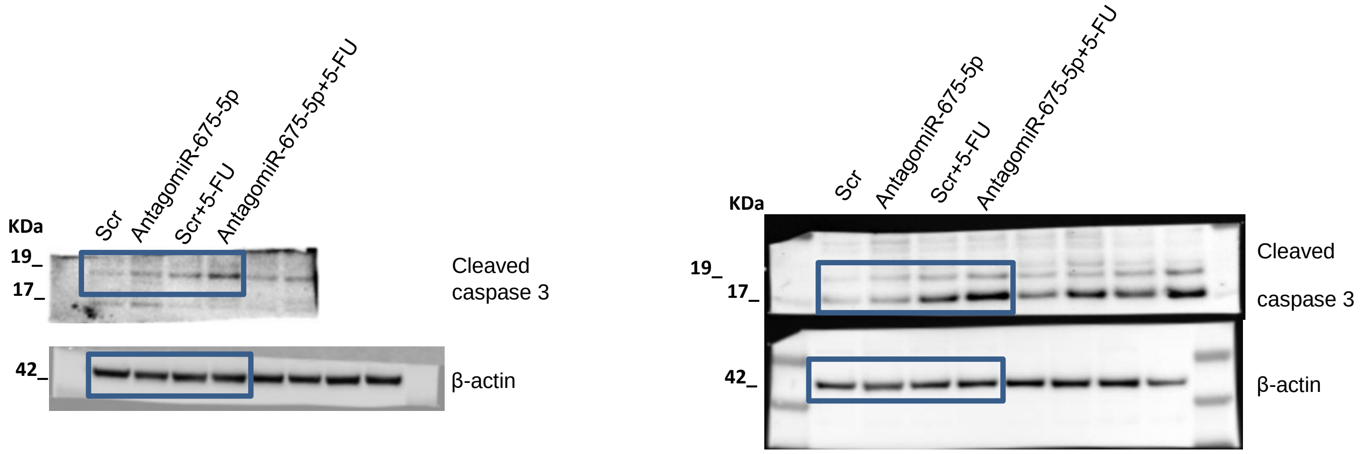

The original uncropped western blot for cleaved PARP/PARP (A), cleaved caspase 3 (B),and b-actin obtained from protein lysates of HCT116 and SW480 in chronic hypoxia, transfected with AntagomiR-675-5p or Scrambled Negative Control (Scr) and treated or not with 5-FU (10µM).

Full-length blot of figure 5

C **HCT116**

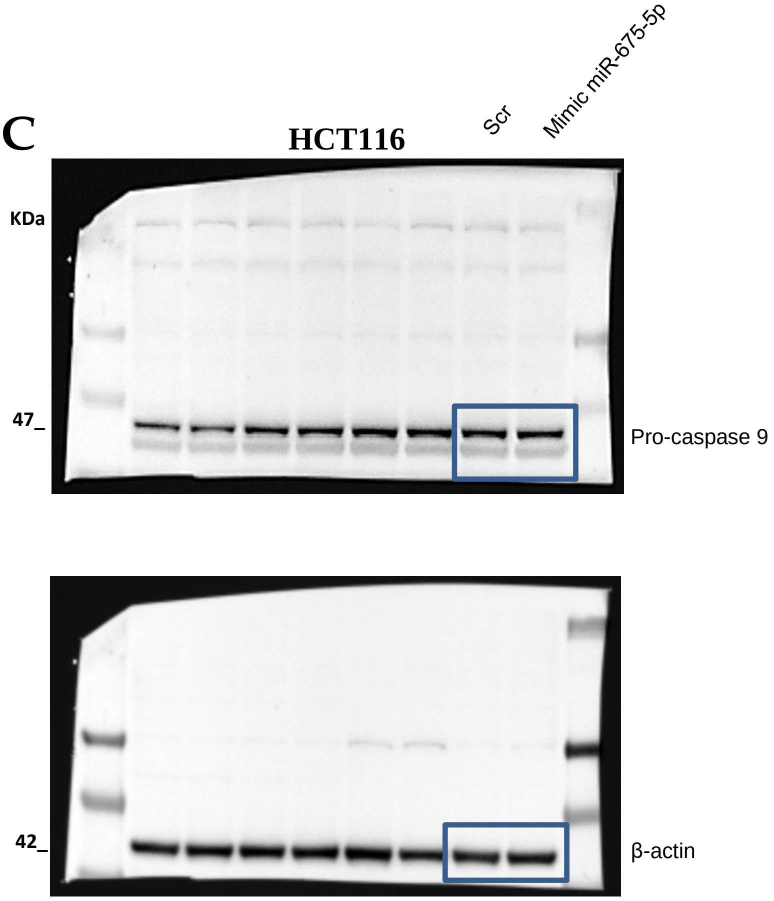

D **HCT116**

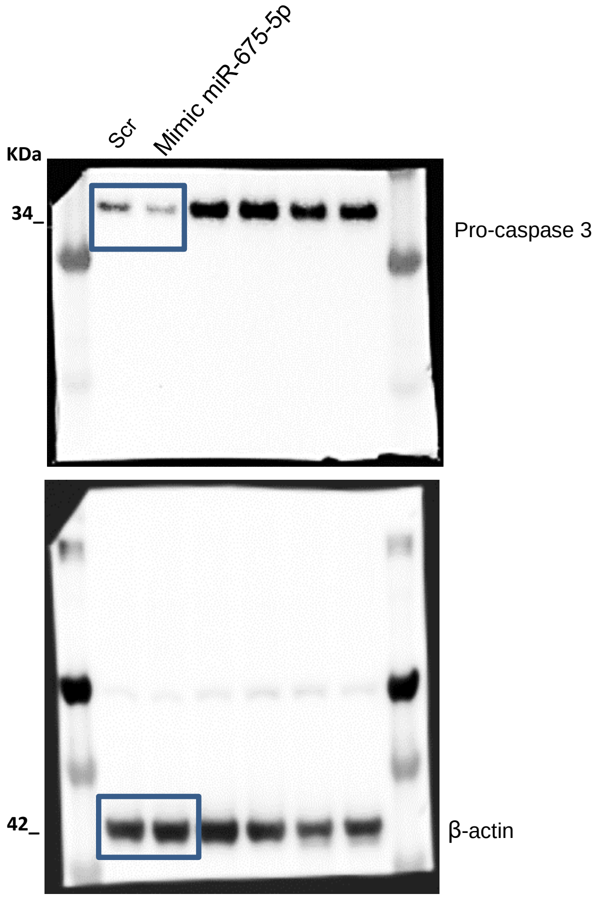

The original uncropped western blot figure for pro-caspase 9 (C) or pro-caspase 3 (D) and b-actin on proteins lysates from HCT116 transfected with miR-675-5p mimic or Scrambled Negative Control (Scr)

Full-length blot of figure 6

A **HCT116**

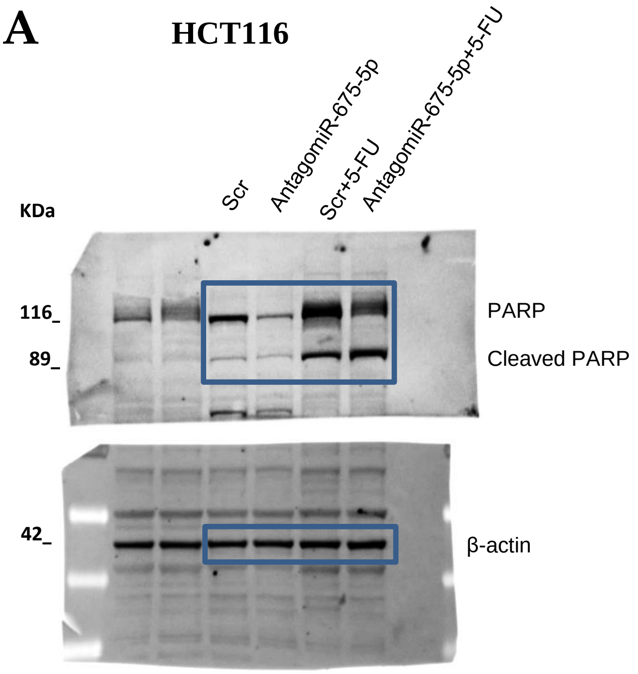

B **HCT116**

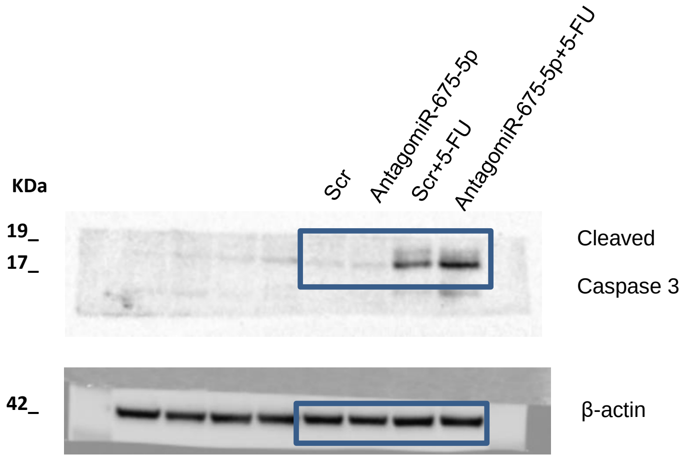

The original uncropped western blot figure for cleaved PARP/PARP (**A**), cleaved caspase 3 (**B**), and b-actin on proteins of the total extract from HCT116 in normoxic conditions, transfected with AntagomiR-675-5p or Scrambled Negative Control (Scr) and treated or not with 5-FU (10µM).
